# A new cylindrical borehole detector for radiographic imaging with muons

**DOI:** 10.1038/s41598-021-96247-1

**Published:** 2021-08-31

**Authors:** L. Cimmino, F. Ambrosino, A. Anastasio, M. D’Errico, V. Masone, L. Roscilli, G. Saracino

**Affiliations:** 1 Department of Physics, University of Naples “Federico II”, Naples , Italy; 2 Istituto Nazionale di Fisica Nucleare, Sezione di Napoli, Naples, Italy

**Keywords:** Applied physics, Imaging techniques, Experimental particle physics

## Abstract

Muon radiography is a methodology which enables measuring the mass distribution within large objects. It exploits the abundant flux of cosmic muons and uses detectors with different technologies depending on the application. As the sensitive surface and geometric acceptance are two fundamental parameters for increasing the collection of muons, the optimization of the detectors is very significant. Here we show a potentially innovative detector of size and shape suitable to be inserted inside a borehole, that optimizes the sensitive area and maximizes the angular acceptance thanks to its cylindrical geometry obtained using plastic arc-shaped scintillators. Good spatial resolution is obtained with a reasonable number of channels. The dimensions of the detector make it ideal for use in 25 cm diameter wells. Detailed simulations based on Monte Carlo methods show great cavity detection capability. The detector has been tested in the laboratory, achieving overall excellent performance.

## Introduction

Muography is a methodology of imaging the interior of a body that takes advantage of the high penetration capacity of cosmic muons^1^. Two methods are currently in use that exploit different properties of interaction of muons with matter. The first is based on the scattering that muons undergo when they pass through high density materials^2–4^; it can be used to identify radioactive materials inside barrels or containers. The muon trajectory is reconstructed at the entrance and exit of the scanned object, whose size is typically less than about 100 m^3^.

The second method is based on the measurement of the absorption of muons; in this case, the number of muons and their direction of arrival are measured. By comparing the attenuated flux with that measured in open sky, a two-dimensional density map of the target volume is obtained. This method permits to image large volumes, even with detectors whose active surface is of the order of square meters. Pioneering works were provided by George^5^ in 1955, who sensed its potential and obtained a measurement of the overburden of a tunnel, and by Alvarez^6^, in the late 1960s, which analyzed the interior of the Chefren pyramid in search of hidden cavities. More recently, muon radiography by absorption has been used for the study of the pyramid of Cheops (Kufhu)^7^, for the discovery of cavities inside a hill^8,9^, or for the exploration of archeological site^10^. For a review of possible applications see^11^.

Some archaeological applications require a detector that can be inserted into a well; the cylindrical detector described in^12,13^, which has a diameter of 14 cm and is made with scintillating fibers and plastic scintillator bars, meets the requirement. Instead, applications for mining exploration purposes^14^ or for the monitoring of the carbon dioxide trapped in the subsoil^15–17^, use detectors with flat-faced plastic scintillators. Both technological solutions have some advantages and some drawbacks.

The use of scintillating fibers guarantees excellent spatial resolutions and is suitable for cylindrical geometries. But, the number of fibers and channels can become prohibitive in the case of large detectors of this type, with a significant increase in the production costs. On the other hand, the use of a planar geometry made of orthogonal scintillator bars with rectangular section, lowers costs but is not very efficient for use inside wells; planar geometries don’t optimize the active surface and the angular acceptance.

The detector presented here uses a novel approach that takes advantage of plastic arc-shaped scintillators, optimizing the performances of a well detector.

## Detector description

The main reason the detector was designed and built is to fit inside a borehole. The diameter of the well is always an important parameter to take into consideration; large wells present logistical difficulties and higher costs. The making of a drilled well, on the other hand, tends to prefer *small* diameters. The prototype of the detector has a diameter of 24 cm, so it can be inserted into a well with a diameter greater than 25 cm; such drilling that can be carried out at a reasonable cost with ordinary drilling machines.

The detector is made of plastic scintillators (DETECT-Europe UPS-932A) optically coupled to Silicon photomultipliers (SiPMs). Plastic scintillators are the elements sensitive to the passage of muons. The detector is equipped with scintillators of two different shapes; one type is in the shape of an arc and another is a common bar, both of which have a rectangular section.

**Figure 1:**
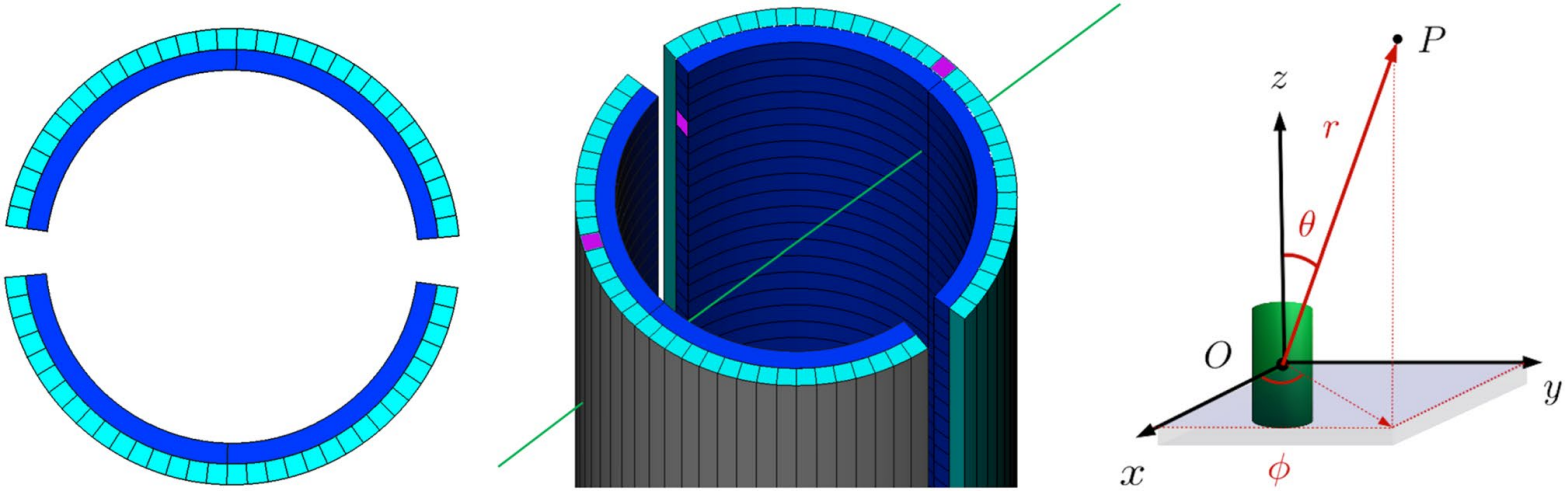
Schematic arrangement of the scintillating elements of the cylindrical detector and an example of a muon passing through the detector illuminating (in violet) its scintillators. On the right, the spherical coordinate system used for tracking.

By considering a polar coordinate system with the *Z*-axis oriented along the cylinder axis (on the right in Fig. 1), the arcs are arranged concentrically with respect to the *Z*-axis and form a cylindrical surface providing the Z coordinate of the impact point of the muon. The bars are instead arranged parallel to the cylinder axis, forming a second cylindrical surface external to the first, and give the azimuthal coordinate $\phi$.

The detector is built by assembling two semicylinders, each composed by 32 bars and 128 arcs. The bars have a rectangular section of $8.5~\text {mm}\times 6.5~\text {mm}$ and are 1 m long. Every arc subtends an angle of about $83^\circ$; the internal radius is 83 mm and the external radius 93 mm and the height is 15 mm. The total number of arcs is 256, that of bars 64, for a total of 320 scintillators elements. The bars are coupled to SiPMs at both ends. The arcs are coupled only to one end, while the other end is covered with aluminum tape, to increase the light collection and avoid the cross talk between two arcs facing each other. The total number of SiPMs is therefore 384.

Great attention has been paid to the transmission of scintillation light along the scintillators, given the fact that arcs introduce a curve in the photon path and that the bars are significantly longer than the arcs. Typically, wavelength shifting fibers embedded in the scintillators guarantee a good light transmission^18^, but we preferred a direct coupling between the photosensor and the scintillator to reduce costs and simplify production; scintillators are used as light guides, with overall excellent performance as shown in^19^. Their surface are carefully polished and the scintillators are lodged in a dedicated rack with dedicated housings for each type of scintillator.

The housing gives the correct mechanical positioning of the scintillators; since the housing guarantees the optical insulation between nearby scintillators, no cover materials have been used to wrap the scintillators. As shown in^19^, a reflective coating slightly improves the scintillator’s light-collection efficiency; but, it makes the detector construction more complex. The internal walls of the rack have thin spacers which ensure that there is a layer of air between the scintillator and the housing itself. In this way the critical angle of $42^\circ$ allows good light transmission along the curve path and the 1 m long bar by means of the total internal reflection, considering that the typical bulk attenuation length of the scintillator is in the range 2–4 m.

**Figure 2:**
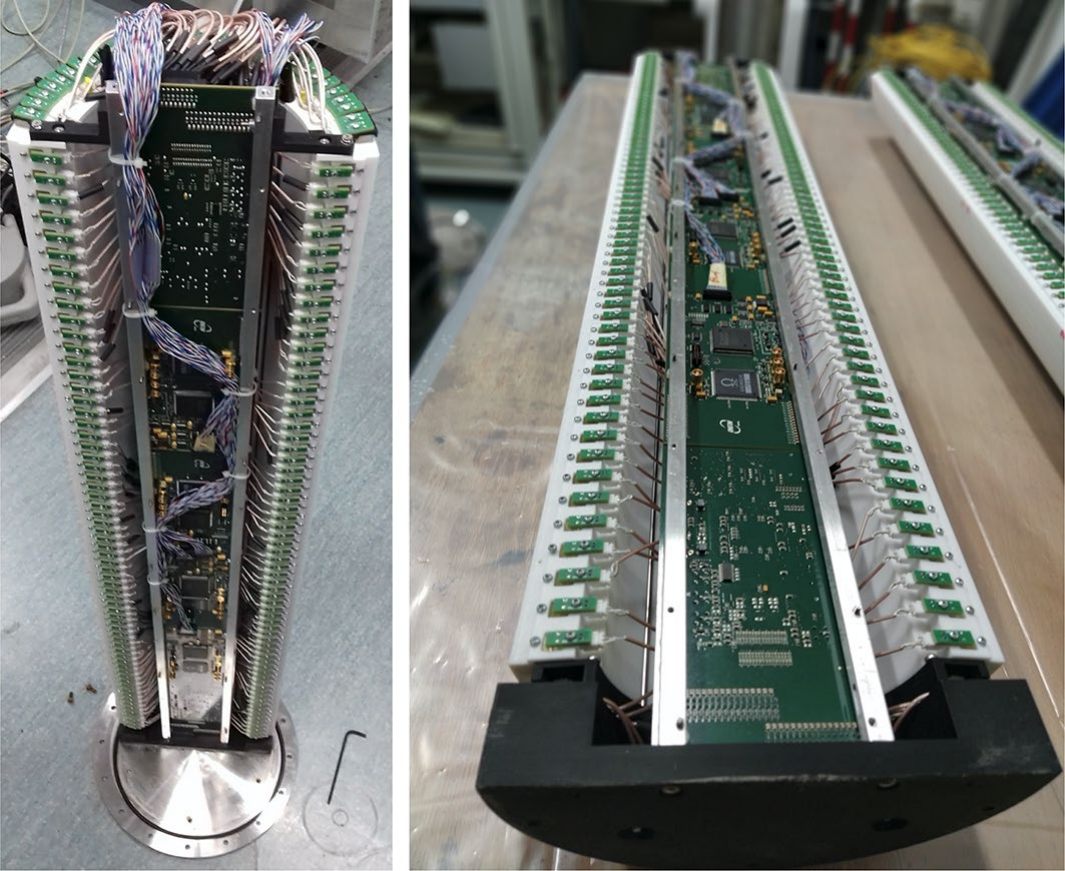
One semicylinder assembled with all the scintillators inserted and the silicon photomultipliers wired to the front end electronics boards.

The supporting structure in Acrylonitrile Butadiene Styrene (ABS) is produced with a 3D printer; it is made up of 8 rack elements, each 1 m long semicylinder is obtained by stacking 4 racks. The front end electronics is housed in its concavity as can be seen in Fig. 2.

The detector is equipped with the SiPM S13360-3050PE manufactured by Hamamatsu. It is a surface mount sensor, with a $50~\upmu \text {m}$ cell pitch and an active area of $3 \times 3~\text {mm}^2$, that is about a tenth of the section surface of the scintillators. Although in theory, to increase the number of collected photons it would be desirable to have the scintillator surface covered by the photosensor as much as possible, the occurrence of the dark counts limits the choice of the size of the SiPM photosensitive area. Furthermore, the price of the single device is proportional to the active surface. For these reasons it is better to limit the sensitive area of the SiPM to that strictly necessary to obtain the desired performance. As tested in laboratory measurements^19^ the coupling between the photosensors and the scintillators produces enough photoelectrons with a reasonable number of dark counts. The photosensor is soldered onto a dedicated printed circuit board (PCB) and connected to a two-pin connector mounted on the same PCB.

The front-end and data acquisition electronics, as described in^20^, are characterized by low power consumption (about 30*W* for the whole detector), modularity and outdoor use capability. The front-end electronics (FEE) boards are equipped with the EASIROC chip^21^ developed for the SiPM readout. The chip has 32 input channels and can store the analog information relating to the number of photons released in each of the scintillators, after a pre-amplification and shaping of the signal, using a sample and hold technique. Moreover, the chip produces a fast logical signal, called Local Trigger (LT), when at least one of the 32 inputs exceeds a settable threshold level.

When a muon passes through the detector, a total of 12 SLAVE boards collect the output of the 384 SiPMs coupled to the scintillating elements. The LT signals produced by all the FEE boards are sent to the MASTER board which, using a programmable logic, produces a global trigger (GT) logical signal, that is sent to all the FEE boards. The GT signal starts the conversion in digital format of the analog information stored in the EASIROC and the data are transferred from the FEE boards to the MASTER board. An ARM single-board computer is embedded in the MASTER board, allowing for DAQ control and data transfer.

Four sensors (HDC1050 by Texas Instruments) are distributed inside the detector and provide temperature and relative humidity measurements in digital format using the $\text {I}^2\text {C}$ protocol; the sensors are read and logged by a single-board microcontroller. The MASTER board and related compute modules are mounted on the top of the detector.

The assembly of the detector is carried out by first mounting the arcs of scintillators and the SiPMs in each of the 8 racks, which are then arranged to form two distinct semicylinders. The 68 scintillating bars, the relative photosensors and the 12 front end electronic boards with connection cables are then inserted. The two semicylinders are then fixed on a stainless steel base, on which a stainless steel cylinder with flanges is then lowered and fixed at the base. The MASTER board is then fastened to the top of the cylinder and wired. A stainless steel cap equipped with two IP68 class connectors, one for the power supply and the other for ethernet communication, definitively closes the detector. Two O-rings are placed on the flanges for waterproofing the detector.

## Detector commissioning

The choice of an optimal working point plays a central role in the commissioning of the detector. To do that we proceed to set the SiPMs bias voltage to the operation voltage as in the specifications (typical bias voltage is 56 V, corresponding to an over-voltage of 3 V and a gain about $1.7 \times 10^6$). We already mentioned that, each EASIROC chip collects the signals of 32 SiPMs and produces the digital LT signal if at least one of the inputs exceeds the common discriminator threshold level. This is set by a programmable digital register called DAC10. The LT frequency is a function of the discriminator threshold level. For low threshold levels the frequency is dominated by dark noise. This measure allows to know the average number of photoelectrons that corresponds to each threshold level of the discriminator.

By determining the threshold levels corresponding to a given cut in terms of photoelectrons, we set the optimal working point for each FEE board. In any case, the performance of SiPMs may vary due to external temperature conditions; in this case, for temperature variations of the order of a few Celsius, we modify the SiPM operating voltages in order to compensate for the variation of the breakdown voltages.

**Figure 3:**
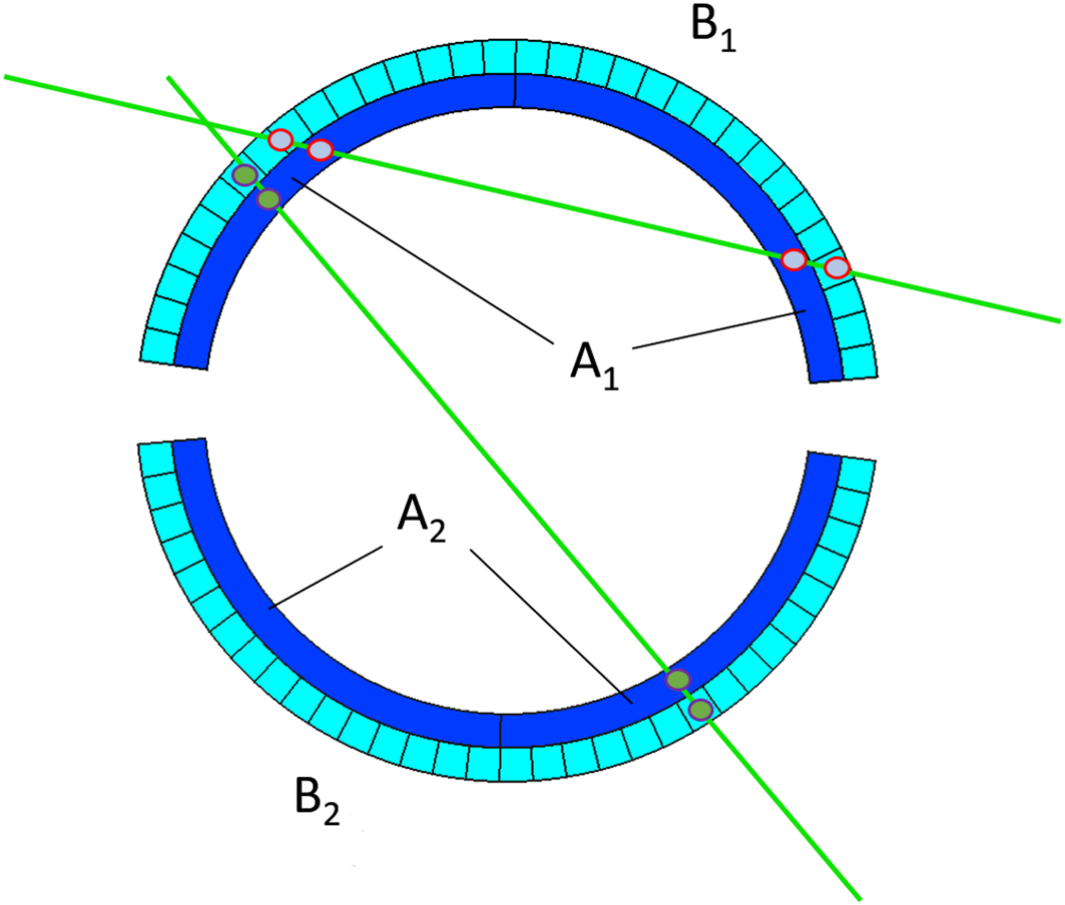
Schematic top view of the detector. The picture shows possible tracks corresponding to the passage of muons.

Track recording is done concurrently with the generation of the GT, and it is produced as follow. Considering one of the two semicylinder, we denote by $B_1$ ($B_2$) the logic signal produced by the passage of a muon in at least one of the 32 bars and with $A_1$ ($A_2$) the logic signal produced by at least one of the 128 arcs of the semicylinder. The detector trigger logic is set to satisfy the logical condition: $\text {GT} =(A_1\wedge B_1)\vee (A_2\wedge B_2)$. In this way, after the processing of the data collected with this trigger logic, as it can be seen in Fig. 3, tracks correspond to muons that have crossed both semicylinders or to muons that have crossed the same semicylinder in two points, thus lighting up two bars and two arcs.

SiPMs can be activated both by the light produced by the passage of muons through the scintillators and by dark counts; there is no difference in satisfying the trigger logic and when the dark counts rate is high, these are very likely to result in accidental quadruple coincidences. To have an estimate of the accidental rate, in a double coincidence, we use the formula $R_{Acc}=2(LT_{A_1}LT_{B_1}+LT_{A_2}LT_{B_2})\tau$, where $\tau$ is the coincidence time window^22^. For a time windows $\tau$ of 100 ns, and LT rates of order 10 KHz we expect an accidental rate of tens of KHz, which would dramatically affect the trigger rate measurement. Therefore, the detector is run at LT rates of hundreds of Hz so that the rate of accidental coincidences, which would turn out to be on the order of $10^{-2}$ Hz, is absolutely negligible.

**Figure 4:**
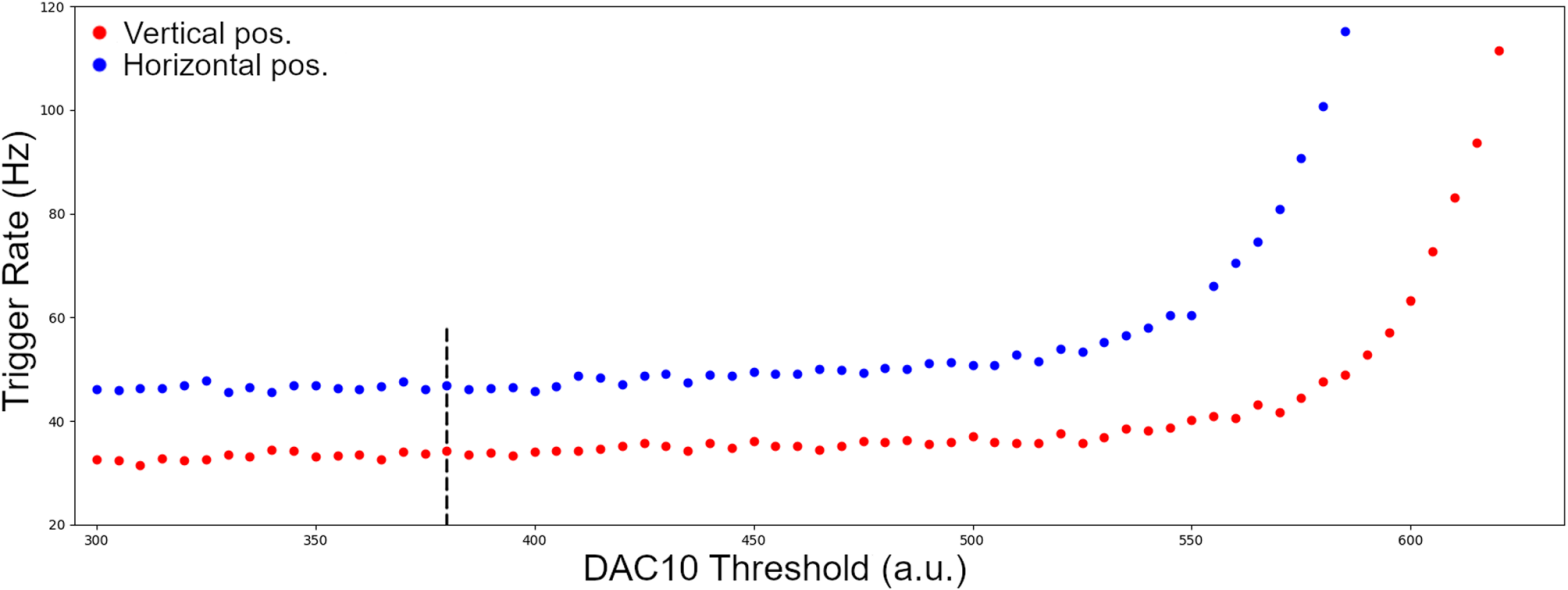
The trigger rate as a function of the discriminator threshold. High value of the DAC10 register corresponds to lower values of the discriminator. The dashed line indicates the threshold value corresponding to a cut-off value of 5–6 photoelectrons.

Figure 4 shows the GT rate, in two different configurations, obtained by changing simultaneously the threshold of the discriminator that produce the LT signal in each FEE board. The threshold values in the plot are shown in arbitrary units and correspond to the setting of Digital-to-Analog converters (DACs). Any DAC value can be related to the equivalent number of photoelectrons using the dedicated dark count calibration measurements. The trigger rate is composed of cosmic particles and accidental coincidences. By increasing the threshold value, the accidental coincidences decrease and a plateau, due to cosmic particles, is observed. We tested the trigger logic $(A_1\wedge B_1)\vee (A_2\wedge B_2)$ with the detector placed vertically and horizontally; plateau values of 37 Hz and 48 Hz was respectively measured at the threshold value of 380, corresponding to a cut-off value of 5–6 photoelectrons.

The acquisition of the dark noise spectrum allows to evaluate the calibration conversion factor for the single photoelectron in terms of ADC counts acquired by the FEE, the so-called single photoelectron response (SPER) and it has been measured to be about 25 ADC counts.

**Figure 5:**
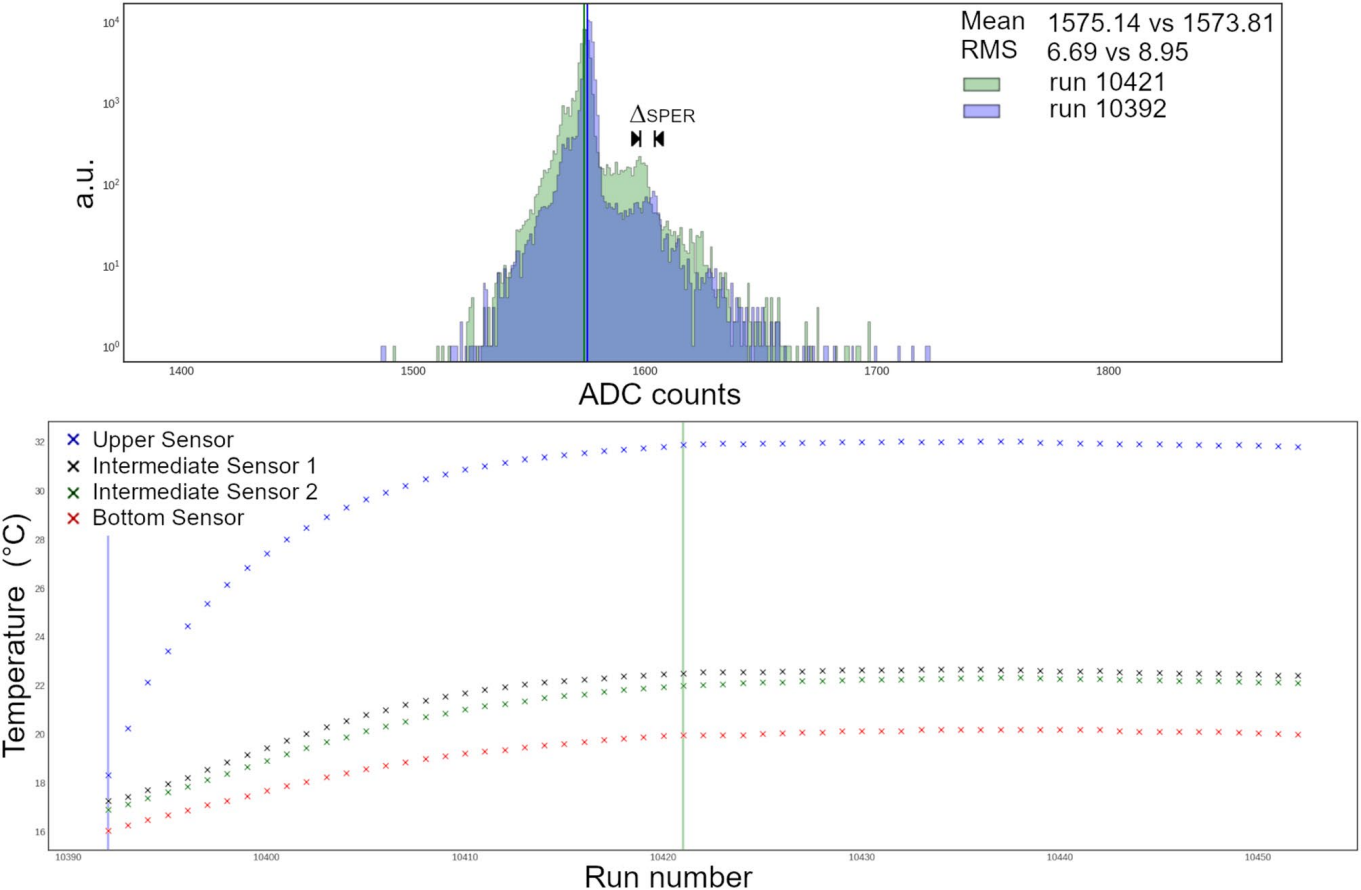
Comparison of two dark noise spectra of a SiPM. The blue spectrum refers to the first test run and the green spectrum to a run in which the temperature curves inside the stainless steel shell have reached the plateau. The increase in temperature inside the detector shell due to overheating of the electronics causes a slight increase in RMS together with a peak value variation of the first photoelectron ($\Delta _{SPER}$). The incremental trend of temperatures was measured by four temperature sensors with the detector in vertical position. Temperatures stabilizes in 5–6 h and the room temperature varies slightly around 15.5 °C.

One week long tests were performed for both vertical and horizontal configuration. Trends in the trigger rate and the LT rates were measured and found to be stable, and the SPERs comparison (Fig. 5) shows a negligible variation in SiPMs performance. The temperatures measured inside the detector by the four temperature sensors when the environmental temperature is about $20^\circ$, tend to increase (Fig. 5) due to the overheating produced by the power dissipation of the electronics, and stabilizes in $\sim 6 \,\text {h}$.

We finally found that the average number of photoelectrons produced in the scintillating arcs and bars decreases as a function of the distance to the end where the SiPM get the reading. If compared to the working point threshold relative to 5–6 photoelectrons, we expect a very high detection efficiency.

**Figure 6:**
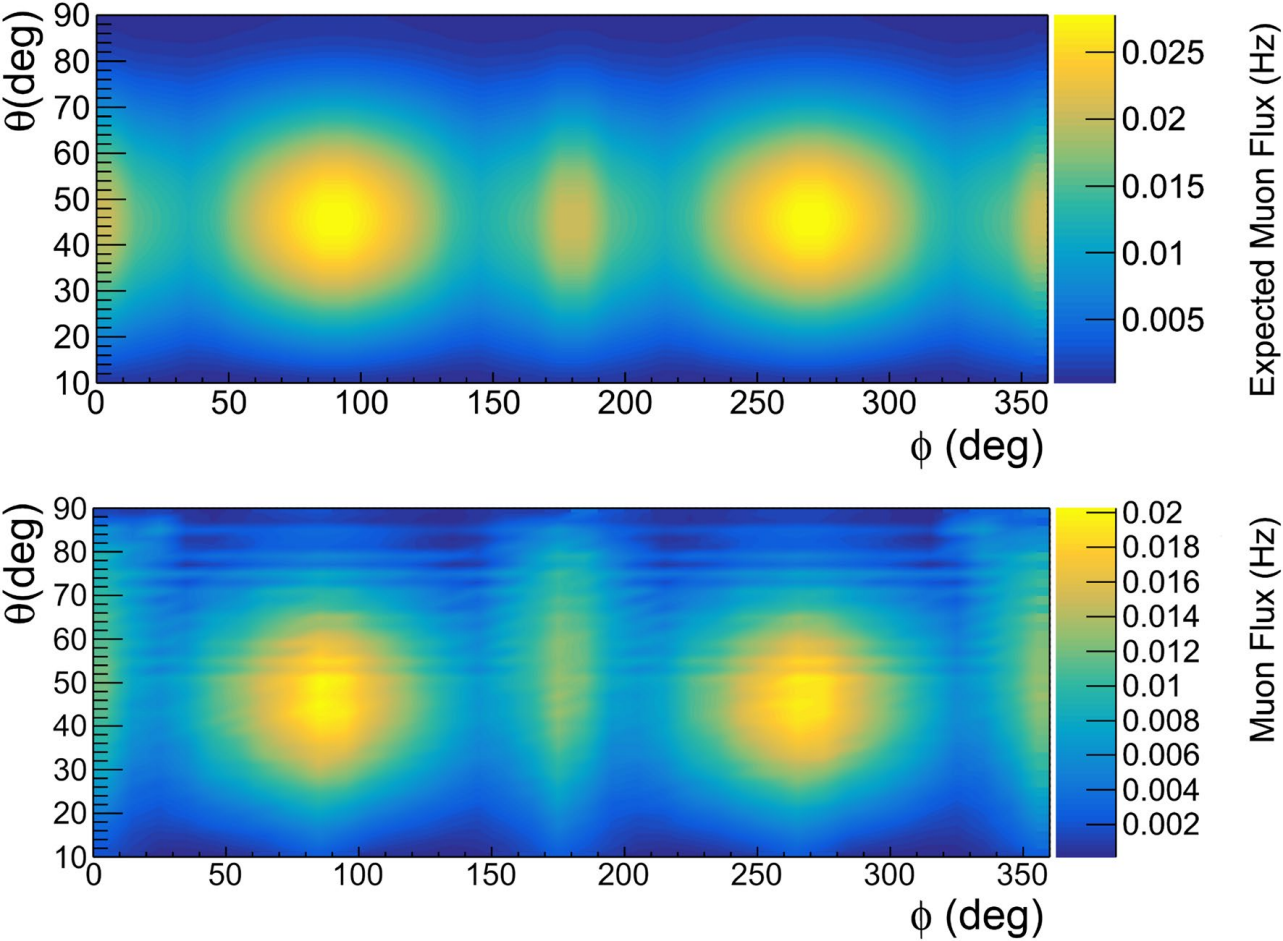
Expected (top) and measured (bottom) free sky rates with the detector placed vertically; the plots are obtained using the software ROOT and the smoothing tool Contour4 is applied. Due to the very low geometrical acceptance, the zenith angular region between $0^\circ$ and $10^\circ$ is not shown.

We acquired a free sky cosmic data sample with the detector placed vertically. The measured angular distribution of the tracks is shown in Fig. 6; in the same figure is shown the expected distribution obtained with synthetic data. The two distributions are in good agreement.

## Expected performance of the detector

The expected performance of the detector was studied using synthetic data produced via the Monte Carlo technique; the *GEANT4*^23^ toolkit was used to simulate the passage of muons through matter and the detector. The use of synthetic data allows us to develop and test the algorithms necessary for data analysis and to compare the performance of the *real* detector with that of an *ideal* detector, with the aim of determining the efficiency and space resolution that best suits this Muon Radiography application.

**Figure 7:**
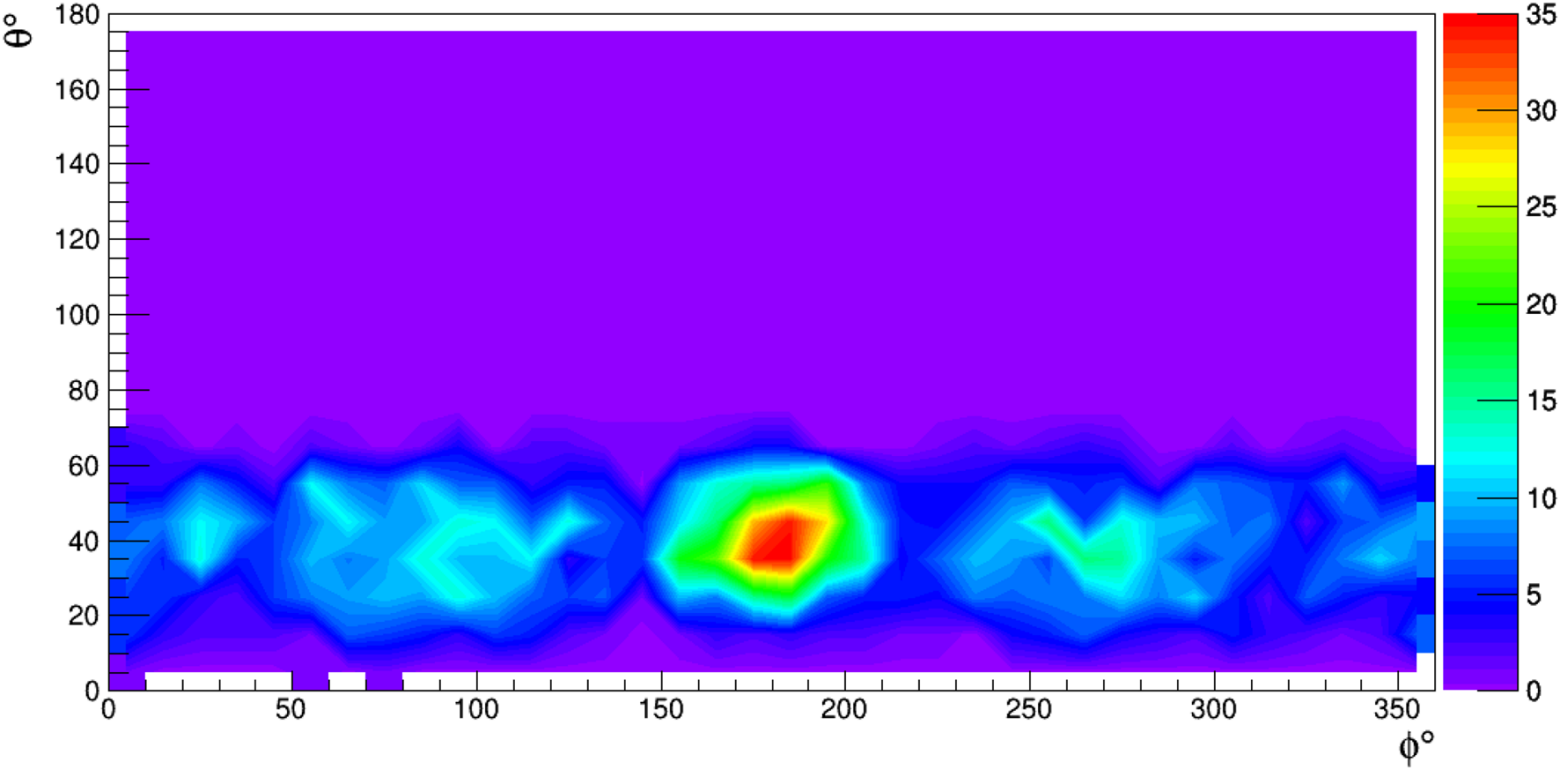
Number of muons (in arbitrary units) as a function of the zenith angle $\theta$ and azimuthal angle $\phi$ observed by the detector in the case of a cavity of 15 m radius, located at a depth of 20 m with the detector at depth of 40 m and a distance of 25 m in the horizontal plane. The center of the cavity has azimuth $180^\circ$.

We developed several algorithms to test detection and localization of a cavity. Some of these algorithms have been included in a patent^24^. In Fig. 7 is shown an example of a cavity of 15 m radius, located at a depth of 20 m with the detector at depth of 40 m and a distance of 25 m in the horizontal plane.

The center of the cavity has a azimuthal angle of $180^\circ$. The plot shows the number of muons passing through the detector, as a function of their angular variables. The presence of the cavity is highlighted by an excess of the number of muons observed in the direction corresponding to the cavity (red zone), due to the smaller amount of material encountered by muons along this direction. The image obtained with the use of simulated data is equivalent to that which would be obtained in a real scenario with an exposure time of about 6 h, with the gap between the 2 semicylinders pointing to the center of the cavity.

## Conclusions

We have described a potentially innovative tracker with small size and cylindrical shape for the borehole applications of Muon Radiography. The detector was made with arc-shaped plastic scintillators, in order to optimize the sensitive surface, directly coupled to Silicon photomultiplier. This simplifies the production of the detector and lowers costs. In particular, much attention was paid to the transport of the scintillation light along the plastic scintillators. The detector is housed in a watertight stainless steel shell, which shields it from alpha and beta particles deriving from natural background radiation, and has low power consumption, so that it can be used in harsh environment. Gamma rays from natural background radioactivity can pass through the steel shielding but are unlikely to produce 4 hits that are needed to define a track. The detector performs well at internal temperatures up to 45 °C and is insensitive to magnetic fields. Detailed Monte Carlo simulations performed with *GEANT4* showed excellent cavity detection capability. The detector was tested in the laboratory with overall excellent performance and a large plateau of cosmic muon counts was measured. The detector and some algorithms tested with synthetic data have been patented.

## Methods

**Figure 8:**
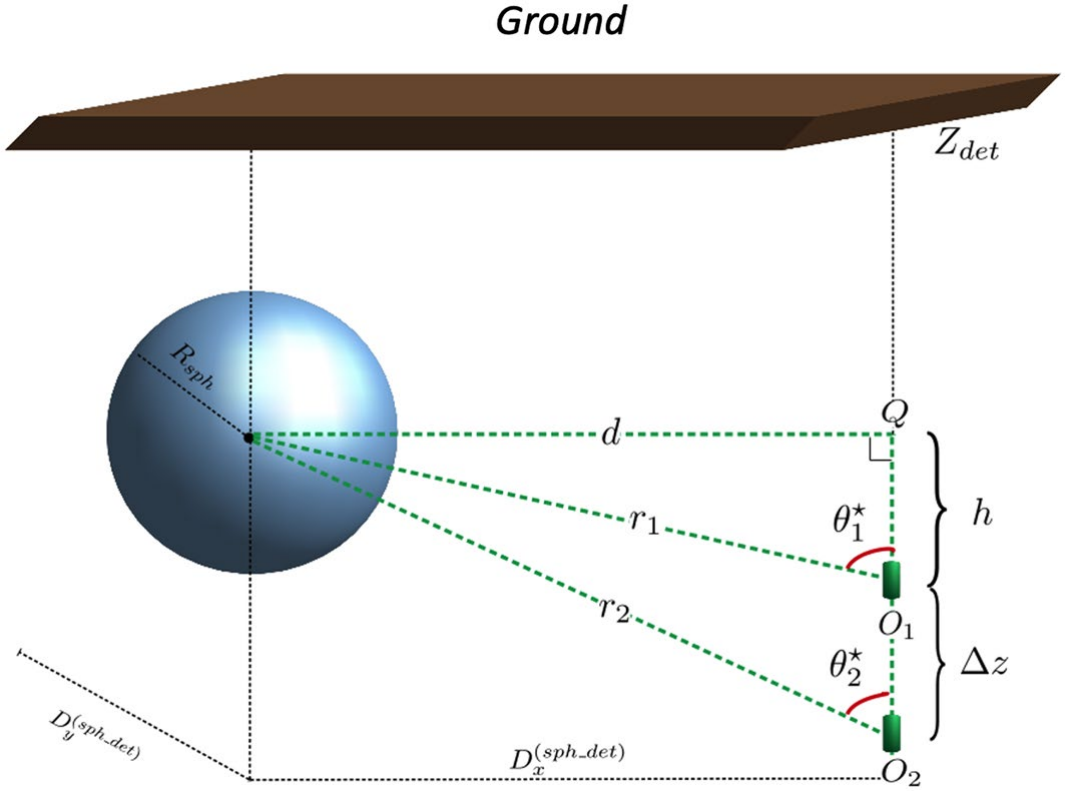
Schematic representation of the *world* volume defined in the GEANT4 simulation tool. The picture also shows the detector at different depths, as used in a triangulation method.

The feasibility study is performed in two different phases; first we produce data similar to what would be expected in a real geophysical scenario, then the data produced are analyzed. The simulated scenario is a *world* with the shape of a parallelepiped, filled with a homogeneous material inside which we place a particle detectors and one (or even more) volumes, which represents a discontinuity in the mass density (see Fig. 8). The top face of the parallelepiped is at the level of the Earth’s surface, on which muons impinge according to the known energy spectra^25^. The density anomaly is modeled as a sphere with a certain radius and uniform density, different from that of the *world*, placed at a certain depth in the ground.

The *real* detector is modeled as in reality. The *ideal* detector, on the other hand, is represented by a hollow cylinder with the same size of the *real* detector but with no dead zones and no discrete segmentation of the sensitive area; the impact point of the muon is determined without errors. Several simulated measurement campaigns were carried out by changing the position and size of the sphere and positioning the detector vertically in different points.

**Figure 9:**
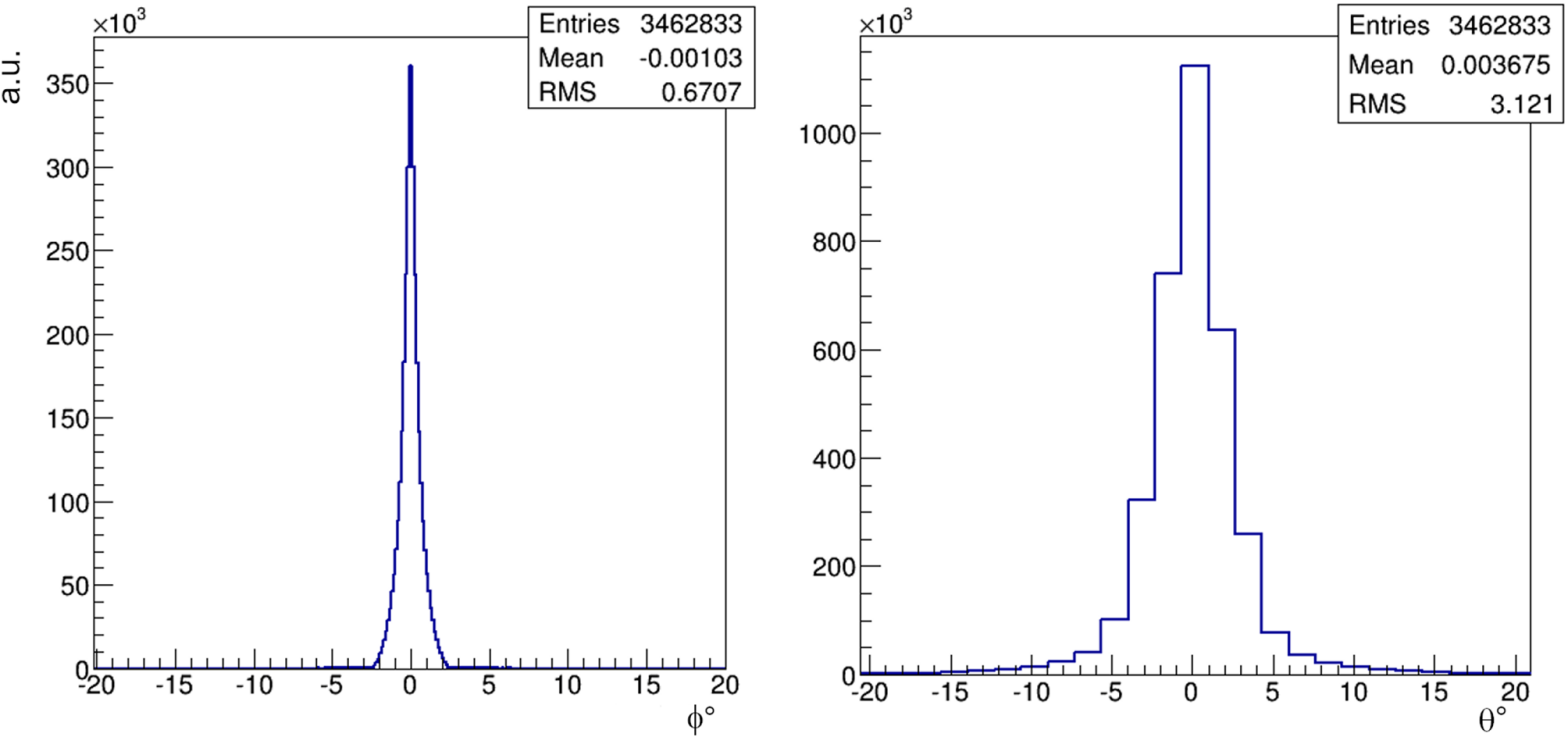
Expected average angular resolutions of the reconstructed azimuth (left) and zenith angle (right).

The Monte Carlo simulation campaigns with the *Geant4* software are very accurate but, to produce high statistics campaigns, large computing resources (CPU time and data storage) are needed. The simulations were performed by the supercomputing infrastructure *SCoPE* of the University of Naples *Federico II*. As it can be seen in Fig. 9, the expected average angular resolutions of the *real* detector, evaluated using the synthetic data, are of order $0.67^\circ$ and $3.1^\circ$ for the azimuth and zenith angle, respectively.

A single detector location gives the direction of the cavity. An estimate of the distance can be obtained with the triangulation technique and can be carried out either using data acquired from two different drilling operations, or using the same well but carrying out measurements at different depths. The latter case is of particular interest because exploiting the same well for different measurements is a significant logistical advantage.

The Fig. 8 shows a pictorial representation of measurements with the triangulation method. The cavity, with a radius of 15 m and whose center is at a depth of 25 m, begins to be visible as soon as part of the empty volume is in the angular acceptance of the detector. From the measurement of the $\theta _1^*$ and $\theta _2^*$ zenith angles of the center of the cavity and knowing the depth at which the detector is placed, using simple geometrical considerations we estimate the distance *r* of the cavity with respect to the detector.
